# *Suillusgrevillei* and *Suillus luteus* promote lead tolerance of *Pinus tabulaeformis* and biomineralize lead to pyromorphite

**DOI:** 10.3389/fmicb.2024.1296512

**Published:** 2024-05-09

**Authors:** Kang Cheng, Yaqin Liu, Ming Tang, Haoqiang Zhang

**Affiliations:** ^1^College of Forestry, Northwest A&F University, Yangling, China; ^2^State Key Laboratory of Conservation and Utilization of Subtropical Agro-Bioresources, Guangdong Laboratory for Lingnan Modern Agriculture, College of Forestry and Landscape Architecture, South China Agricultural University, Guangzhou, China

**Keywords:** ectomycorrhizal fungi, photosynthetic pigment, antioxidant capacity, XRD analysis, biomineralization

## Abstract

Lead (Pb) is a hazardous heavy metal that accumulates in many environments. Phytoremediation of Pb polluted soil is an environmentally friendly method, and a better understanding of mycorrhizal symbiosis under Pb stress can promote its efficiency and application. This study aims to evaluate the impact of two ectomycorrhizal fungi (*Suillus grevillei* and *Suillus luteus*) on the performance of *Pinus tabulaeformis* under Pb stress, and the biomineralization of metallic Pb *in vitro*. A pot experiment using substrate with 0 and 1,000 mg/kg Pb^2+^ was conducted to evaluate the growth, photosynthetic pigments, oxidative damage, and Pb accumulation of *P. tabulaeformis* with or without ectomycorrhizal fungi. *In vitro* co-cultivation of ectomycorrhizal fungi and Pb shots was used to evaluate Pb biomineralization. The results showed that colonization by the two ectomycorrhizal fungi promoted plant growth, increased the content of photosynthetic pigments, reduced oxidative damage, and caused massive accumulation of Pb in plant roots. The structural characteristics of the Pb secondary minerals formed in the presence of fungi demonstrated significant differences from the minerals formed in the control plates and these minerals were identified as pyromorphite (Pb_5_(PO_4_)_3_Cl). Ectomycorrhizal fungi promoted the performance of *P. tabulaeformis* under Pb stress and suggested a potential role of mycorrhizal symbiosis in Pb phytoremediation. This observation also represents the first discovery of such Pb biomineralization induced by ectomycorrhizal fungi. Ectomycorrhizal fungi induced Pb biomineralization is also relevant to the phytostabilization and new approaches in the bioremediation of polluted environments.

## 1 Introduction

Lead (Pb) is a hazardous heavy metal that causes severe environmental and human health problems (Rehman et al., 2017; Monchanin et al., 2021). Pb is mainly introduced into soils by human activities such as lead acid battery production, paint production, mining, and leaded petrol production (Jarup, 2003; Li et al., 2014). Pb pollution restricts soil usage and fertility due to the non-degradable and toxic characteristics of Pb (Adriano, 2001). Phytoremediation is an effective and viable method for Pb polluted soils (Sarwar et al., 2017). However, Pb harms plant development and survival due to the production of an excessive amount of reactive oxygen species (ROS) (Reddy et al., 2005; Zhang et al., 2020). Consequently, phytoremediation has some drawbacks, including sluggish development of the accumulator plants, poor biomass production, and low heavy metal absorption, which makes it a lengthy and inefficient procedure (Vergara et al., 2020).

Ectomycorrhizal (ECM) symbiosis is widespread in numerous ecosystems between fungi from Basidiomycota, Ascomycota, and Zygomycota, and the ecologically and economically most important forest trees, including Pinaceae, Fagaceae, Salicaceae, Betulaceae, Caesalpinioideae, Dipterocarpaceae, and Phyllanthaceae (Tedersoo et al., 2010). ECM fungi form Hartig nets inside plant roots, form sheath-like mantles around lateral roots, and form extrametrical mycelia to explore, absorb, and translocate nutrients and water from the surrounding soil. The ECM symbiosis promotes plant nutrition and water uptake, increases plant growth performance, and facilitates the establishment of host plants in harsh environments (Arocena and Glowa, 2000; Baum et al., 2006; Szuba et al., 2017; Wen et al., 2017; Liu et al., 2020). Microbe-enhanced phytoremediation that using ECM is an effective measure for remediating metal-contaminated soils (Shi et al., 2019; Liu et al., 2020). The ECM symbiosis results in enhanced host plants’ tolerance to heavy metals, including alleviation of inhibition of plant photosynthesis caused by heavy metals and a beneficial impact on reducing the metal-induced oxidative stress on plants (Schützendübel and Polle, 2002; Canton et al., 2016; Fernández-Fuego et al., 2017; Mohammadhasani et al., 2017).

In addition to improving the growth and enhancing the heavy metal tolerance of accumulator plants, ECM fungi may play a role in biomineralization. Biomineralization is the process of living organisms’ induced mineral formation. The majority of fungal-involved biomineralization is the consequence of metabolic processes that affect the external environment in a way that facilitates mineral precipitation. Examples of these processes include changes in pH, O_2_, redox potential, redox transformations of metal species, and excretion of organic and inorganic metabolites like CO_2_, H^+^, or organic acids (Gadd, 2021). Fomina et al. (2007) showed that *Beauveria caledonica* causes uranyl phosphate minerals formation via biomineralization. *Bacillus cereus* 12-2, which was isolated from lead-zinc mine tailings, could transform the Pb into rod-shaped Ca_2.5_Pb_7.5_(OH)_2_(PO4)_6_ nanocrystal (Chen et al., 2016). Povedano-Priego et al. (2016) described lead phosphate formation via biomineralization in the interaction of *Penicillium chrysogenum* with metallic Pb. *Phanerochaete chrysoporium* participates in Pb biomineralization and transforms Pb into Pb_5_(PO_4_)_3_OH through fungal phosphatase (Zhao et al., 2020).

*Pinus tabulaeformis* Carr. is one of the most widely distributed pines in northern China (Chen et al., 2008) and it has remarkable drought endurance and great adaptation to poor soil (Wang and Guo, 2010). The well-known ectomycorrhizal species *P. tabuliformis*, characterized by its strong mycorrhizal dependency and potential colonization by various fungal species (Allen, 1991), can be effectively used for restoration in post-mining areas (Yan et al., 2020; Zhang et al., 2023). It was found that the dominant ECM fungi in the rhizosphere soil of *P. tabuliformis*, which belongs to the genus Suillus (Wang et al., 2022), serves as a model system for understanding mycorrhizal fungal metal tolerance (Branco et al., 2022). Although there were studies showing that pines with ectomycorrhizal fungi promoted tolerance against heavy metals (Bizo et al., 2017; Liu et al., 2020; Ouatiki et al., 2022), the response of the symbiosis between *P. tabulaeformis* and ectomycorrhizal fungi to Pb pollution was rarely reported. In this study, we used two ectomycorrhizal fungi (*Suillus luteus* and *Suillus grevillei*) to evaluate their ability to (1) form symbiosis with *P. tabuleaformis*, (2) improve plant growth, (3) promote the activity of antioxidant enzymes and photosynthetic pigment content, (4) regulate Pb uptake and distribution, and (5) biomineralize Pb *in vitro*.

## 2 Materials and methods

### 2.1 ECM fungi, plant material, and growth substrate

The ECM fungi (*Suillus luteus*, NCBI: txid5384 and *Suillus grevillea*, NCBI: txid5382) were stored in the microbiology lab of the Forestry College, Northwest A&F University. These strains were originally isolated from the ectomycorrhizae of *P. tabulaeformis* in Ningshan county, Shaanxi Province, China. These fungi were grown on Modified Melin-Norkrans (MMN) solid medium (Fomina et al., 2005). After 2 weeks of growth, four blocks of media for each ECM fungus (1 cm in diameter) were inoculated in 300 mL MMN liquid medium. After a shaking culture (25°C, 150 rpm in darkness) of ECM for 21 days, the mycelia were obtained by filtering. The mycelia were washed five times with sterilized water, homogenized by blender with 500 mL sterilized water, and used as inoculum.

The seeds of *Pinus tabulaeformis* were obtained from the Forestry Technology Extension Station of the Forestry Department, Shaanxi Province, China. The seeds were surface disinfected by 0.5% KMnO_4_ for 10 min, washed 3 times with sterilized water, and immersed in sterilized water at 45°C for 1 h. Afterward, seeds were germinated on wet filter papers in Petri dishes at room temperature (20–25°C) in darkness. Germinated seeds were transplanted in seedling trays (50 mL) filled with sterilized vermiculite and cultivated in a greenhouse with a temperature of 20–30°C, a photoperiod of 12 h.

Inoculation of ECM was performed 1 week after the transplantation of the germinated seeds of *P. tabulaeformis*. Inoculation was achieved by injecting 10 mL inoculum to the base of seedling in the seedling tray. The nonmycorrhizal seedlings received autoclaved inoculum. Inoculation repeated 3 times with interval of 2 weeks. The seedlings were daily watered with deionized water and weekly fertilized with 10 mL of Hoagland’s nutrient solution (Zhang et al., 2020). The success of ECM fungal colonization was proven by the observation of mycorrhizal structure under the microscope after 3 months (not quantified).

For the pot experiment, the substrate was a mixture of quartz river sand, vermiculite, and soil in a ratio of 1:1:1 (v: v: v). The soil was collected from the top layer of the Northwest A&F University campus nursery in Yangling, Shaanxi Province, China. The main soil nutrient characteristics were as follows: 16.15 g/kg organic matter, 30.35 mg/kg available nitrogen, 20.40 mg/kg available phosphorus, and 126.36 mg/kg available potassium. The background value of Pb concentration in soil was 15.83 mg/kg. Soil was ground, passed through a 2 mm sieve, and mixed with thoroughly washed sand and vermiculite. The substrate was autoclaved at 0.11 MPa and 121°C for 2 h.

The Pb^2+^ concentration in the substrate was adjusted to 1,000 mg/kg by spraying 100 mM Pb(NO_3_)_2_ solution to substrate according to the risk intervention values for soil contamination of agricultural land (Soil environmental quality-Risk control standard for soil contamination of agricultural land, GB 15618-2018). The corresponding amount of NH_4_NO_3_ was added to the control group to make up for the difference in nitrogen content caused by the addition of Pb(NO_3_)_2_. The substrate was used 1 month after the addition of Pb for equilibration.

### 2.2 Experimental design

The experiment consisted of 2 factors (3 × 2): mycorrhizal status, inoculated with *S. luteus* (SL), *S. grevillea* (SG), or not (CK); Pb status, extra Pb was mixed with the substrate (1,000 mg/kg, Pb-treated) or not (0 mg/kg, Pb-free). Three randomly selected mycorrhizal or nonmycorrhizal seedlings were transplanted in a plastic pot filled with 0.4 kg Pb-treated or Pb-free substrate and cultivated for 4 months. There were 6 treatments, and 3 replicates for each treatment.

The pot experiment was conducted in a greenhouse at a temperature of 20–35°C, a photoperiod of 12–14 h, and a relative air humidity of 55–78%. The plants were daily watered with deionized water and fertilized every 2 weeks with 50 mL of Hoagland’s nutrient solution.

### 2.3 Plant growth and ectomycorrhizal colonization

Four months after the transplantation of seedlings in pots, seedling shoots and roots were harvested separately. Seedling roots were carefully washed with tap water to remove all soil particles and dried with paper towels. The fresh shoot and root weight were recorded. The shoot and root were then randomly divided into 4 parts. One part of the shoot and root was oven dried at 80°C until constant weight and was used to calculate the fresh-to-dry mass ratio. The shoot and root dry weight was calculated according to the fresh weight and fresh-to-dry mass ratio. One part of the shoot and root was oven dried and used to measure the Pb concentration. One part of the shoot was used to measure the photosynthetic pigments content. One part of the root was used to measure the mycorrhizal colonization rate. The rest shoot and root samples were frozen by liquid nitrogen and stored in a refrigerator at −80°C.

The ectomycorrhizal colonization rate was calculated according to the cross-griding method (Püttsepp et al., 2004) after roots were stained with trypan blue (Phillips and Hayman, 1970). Twenty 1 cm root segments were analyzed and a total of 200 intersections were counted for a single replicate.

### 2.4 Determination of Pb concentration, photosynthetic pigments content, and antioxidant capacity and oxidative stress

Pb concentration was analyzed with a microwave mineralizer (Multiwave PRO, Anton Paar, GmbH, Austria) and a flame atomic absorption spectrophotometer (AA7000, Shimadzu, Japan) (Vergara et al., 2020).

Chlorophyll a, chlorophyll b, and carotenoids were determined according to the method described by Lichtenthaler and Wellburn (1983).

Fresh shoots and roots that stored in a refrigerator (−80°C) were ground to powder under liquid nitrogen (three biological replicates) for plant antioxidant capacity and oxidative stress assay. The H_2_O_2_ and MDA level was determined with the trichloroacetic acid (TAC) test and the thiobarbituric acid (TBA) test, respectively, according to Velikova et al. (2000). The enzymatic activity (SOD, POD, and CAT) was calculated as the previous study described (Martins et al., 2011; Fernández-Fuego et al., 2017).

### 2.5 Pb transformation by ectomycorrhizal fungi *in vitro*

In order to assess the Pb transformation by ectomycorrhizal fungi, co-culture of Pb shots (4 mm in diameter) and ectomycorrhizal fungi was carried out in Petri dishes (9 cm in diameter) containing MMN solid medium. The Pb shots were autoclaved and evenly placed on the MMN solid medium, while a block of ectomycorrhizal fungal mycelia taken from the edge of a growing colony was placed on the surface of the MMN solid medium. Dishes that received only Pb shots were used as controls. The Petri dishes were incubated at 25°C in darkness for 2 months.

The elemental composition of secondary minerals formed on the Pb shot surface was analyzed with energy dispersive spectroscopy (EDS, AMETEK, United States). Images of secondary minerals on the Pb shot surface were obtained after the Pb shots were sprayed with gold/palladium using an ion sputter (MC1000, Hitachi, Japan) and then used for SEM analysis by a field emission scanning electron microscope (S-4800, Hitachi, Japan).

The composition of secondary minerals generated on the Pb shot surface was determined using X-ray diffraction (XRD) analysis (Zhao et al., 2020). Mineral phases were identified according to International Centre for Diffraction Data Powder Diffraction File (PDF-4 release 2010).

### 2.6 Statistical analysis

IBM SPSS^®^ Statistics was used for data analysis (SPSS Version 26, SPSS Inc., United States). Two- and one-way analyses of variance (ANOVA) with Tukey’s honest significant difference (HSD) tests at *p* < 0.05 were used to confirm statistical significance (Zhao et al., 2020).

## 3 Results

### 3.1 Plant growth and ectomycorrhizal colonization

After 4 months of pot culture, plant biomass and ECM fungi colonization rate were recorded (Table 1). Under Pb-free condition, inoculation of *S. grevillea* improved the shoots biomass and total biomass, while inoculation of *S. luteus* did not contribute to plant growth. Pb addition decreased the growth of *P. tabulaeformis.* Under Pb-treated condition, the plants colonized by *S. grevillea* showed superiority in shoots, roots, and total biomass compared with nonmycorrhizal plants, while the improvement of plant biomass by *S. luteus* was not obvious.

**Table 1:** Plant growth and ectomycorrhizal colonization of *P. tabuliformis* 4 months after inoculation.

Pb treatment	Fungi	Dry weight of shoot (g)	Dry weight of root (g)	Dry weight of total biomass (g)	Colonization rate (%)
0	CK	0.77 ± 0.06bc	0.59 ± 0.06a	1.36 ± 0.02b	0
	SG	1.13 ± 0.12a	0.69 ± 0.04a	1.82 ± 0.08a	76.67 ± 5.59a
	SL	0.90 ± 0.06b	0.60 ± 0.08a	1.49 ± 0.14b	64.85 ± 1.04b
1,000	CK	0.56 ± 0.02d	0.33 ± 0.02b	0.89 ± 0.03d	0
	SG	0.88 ± 0.04bc	0.59 ± 0.03a	1.47 ± 0.01b	68.60 ± 4.48ab
	SL	0.69 ± 0.07 cd	0.41 ± 0.05b	1.10 ± 0.05c	54.17 ± 1.80c

CK, non-mycorrhizal; SG, *Suillus grevillea*; SL, *Suillus luteus*. Values are presented as means ± SD (*n* = 3). Different letters indicate significant differences at *p* < 0.05 by Tukey’s test.

No mycorrhizal structure was observed in nonmycorrhizal plants. Pb addition decreased the colonization rate of *S. luteus* but not *S. grevillea*. More than 50% of the *P. tabulaeformis* roots were colonized by both ECM fungi, and *S. grevillea* colonized at a greater rate than *S. luteus* under both Pb-treated and Pb-free conditions.

### 3.2 Influence of ECM fungi inoculation on plant Pb absorption and distribution

Pb treatment and two ECM fungi inoculation had a significant influence on plant Pb concentration and content (Table 2). The Pb concentration in plant shoots and roots was significantly increased after Pb addition. Inoculation with *S. grevillea* and *S. luteus* increased Pb concentration in roots and decreased Pb concentration in shoots at Pb-treated condition, which increased 43.6% (SG) and 21.9% (SL, not significant) in roots and decreased 37.1% (SG) and 40.6% (SL) in shoots.

**Table 2:** Effect of ECMF on Pb concentration and content in *P. tabuliformis* roots and shoots.

Pb treatment	Fungus	Shoot Pb concentration (mg/kg)	Shoot Pb content (mg)	Root Pb concentration (mg/kg)	Root Pb content (mg)
0	CK	169.87 ± 12.08d	0.131 ± 0.011c	257.26 ± 12.81c	0.152 ± 0.006d
	SG	254.95 ± 14.87c	0.287 ± 0.010ab	451.44 ± 14.87c	0.314 ± 0.028d
	SL	173.13 ± 20.58 cd	0.155 ± 0.013c	327.65 ± 20.58c	0.195 ± 0.032d
1,000	CK	628.49 ± 24.72a	0.351 ± 0.004a	2390.20 ± 24.72b	0.785 ± 0.111c
	SG	395.18 ± 48.40b	0.348 ± 0.033a	3432.78 ± 48.40a	2.025 ± 0.285a
	SL	373.20 ± 42.45b	0.255 ± 0.004b	2912.94 ± 42.45ab	1.194 ± 0.155b

CK, non-mycorrhizal; SG, *Suillus grevillea*; SL, *Suillus luteus*. Values are presented as means ± SD (*n* = 3). Different letters indicate significant differences at *p* < 0.05 by Tukey’s test.

### 3.3 Photosynthetic pigments content

**Figure 1:**
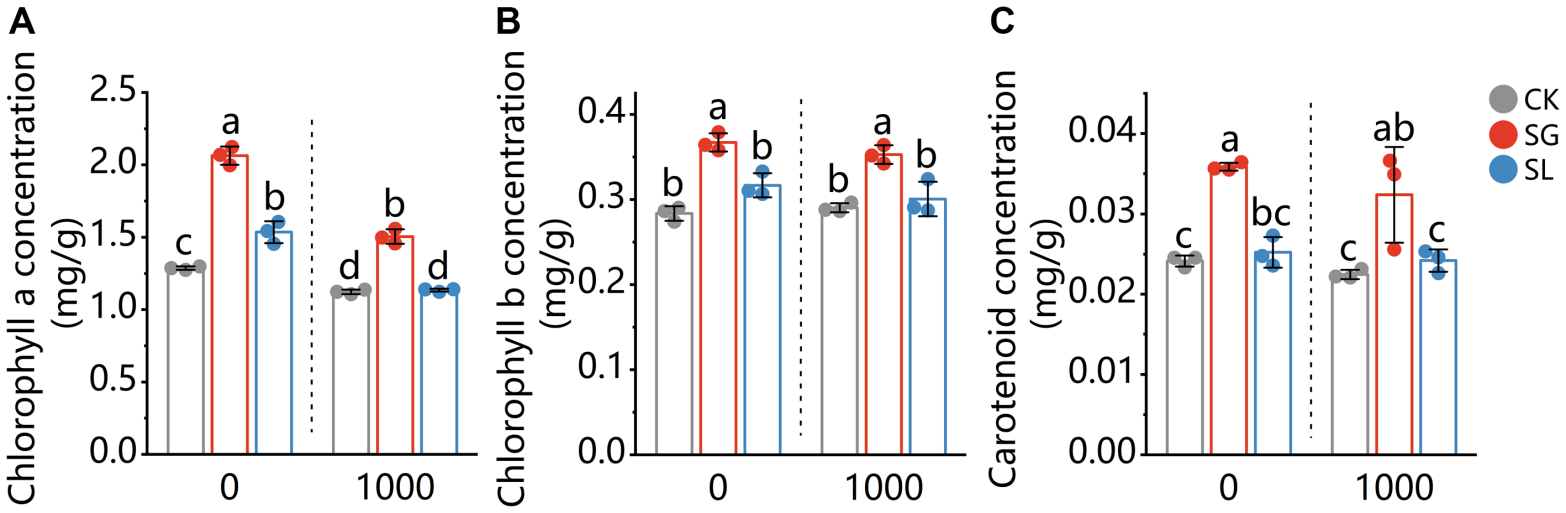
The chlorophyll a **(A)**, chlorophyll b **(B)**, and carotenoid **(C)** content in shoots of *P. tabuliformis*, grown for 4 months in Pb-free treatment (0) or Pb addition (1,000) treatment. CK for nonmycorrhizal and SG and SL for inoculation with *Suillus grevillei* and *Suillus luteus*. Values are presented as means ± SD (*n* = 3). Different letters indicate significant differences at *p* < 0.05 by Tukey’s test.

The chlorophyll a concentration was decreased by Pb addition (Figure 1). Compared with nonmycorrhizal plants, *S. grevillea* inoculated plants had a significant increased chlorophyll a concentration under Pb-free and Pb-treated conditions. For *S. luteus* inoculated plants, a significant increase in the chlorophyll a concentration was observed under Pb-free condition, while there was no superiority after Pb addition. The chlorophyll b and carotenoids concentrations of needles were not decreased by Pb addition (comparing plants grown in Pb-free or Pb-treated conditions). The chlorophyll b and carotenoids concentrations increased in the needles of *S. grevillea* colonized plants.

### 3.4 Antioxidant capacity and oxidative stress

**Figure 2:**
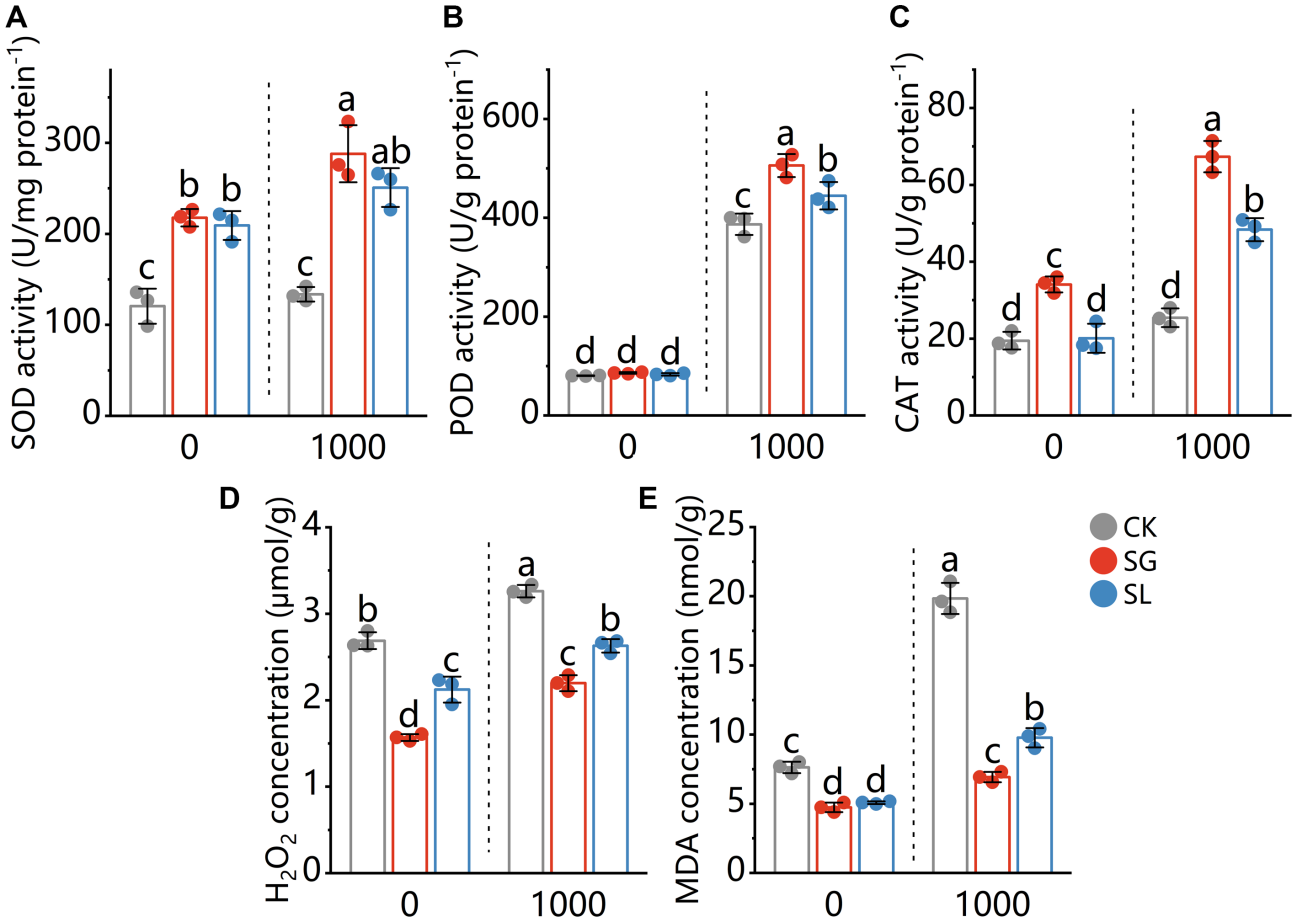
Enzymatic activities of superoxide dismutase (SOD) **(A)**, peroxidase (POD) **(B)**, catalase (CAT) **(C)** and the content of H_2_O_2_ **(D)** and MDA **(E)** in shoots of *P. tabuliformis*, grown for 4 months in Pb-free treatment (0) or Pb addition (1,000) treatment. CK for nonmycorrhizal and SG and SL for inoculation with *Suillus grevillei* and *Suillus luteus*. Values are presented as means ± SD (*n* = 3). Different letters indicate significant differences at *p* < 0.05 by Tukey’s test.

The activity of SOD in plant tissue was not influenced by Pb stress (comparing plants grown in Pb-free or Pb-treated conditions). A considerable rise in SOD activity was observed in the plants inoculated with the *S. grevillea* and *S. luteus* (Figure 2A). Pb stress considerably increased POD activity (comparing plants grown in Pb-free or Pb-treated conditions) in plant tissue. The POD activity in plant tissue further increased significantly for both fungi colonization under Pb stress but showed no difference under Pb-free conditions compared with nonmycorrhizal plants (Figure 2B). The CAT activity in nonmycorrhizal plant tissue was not influenced by Pb stress. Under 2 Pb conditions, a significant increase in CAT activity was observed in the plants that had been colonized by the *S. grevillea*. When exposed to Pb, *S. luteus* colonized plants showed a considerable increase in CAT activity, but not in Pb-free circumstances. CAT activity from *S. grevillea* colonized plants was significantly higher than *S. luteus* colonized plants (Figure 2C).

Pb addition increased H_2_O_2_ concentrations in plant tissue. The H_2_O_2_ concentration in plant tissue was significantly decreased by both fungi (about 41.6 and 48.4% by *S. grevillea*, about 21.0 and 19.4% by *S. luteus* under Pb-free or Pb-treated conditions respectively) (Figure 2D). Pb addition increased the MDA level in plant tissue. The MDA concentration in plant tissue was decreased by both fungi (about 37.8 and 65.1% by *S. grevillea*, about 33.4 and 50.8% by *S. luteus* under Pb-free or Pb-treated conditions respectively) (Figure 2E).

### 3.5 Pb transformation *in vitro*

**Figure 3:**
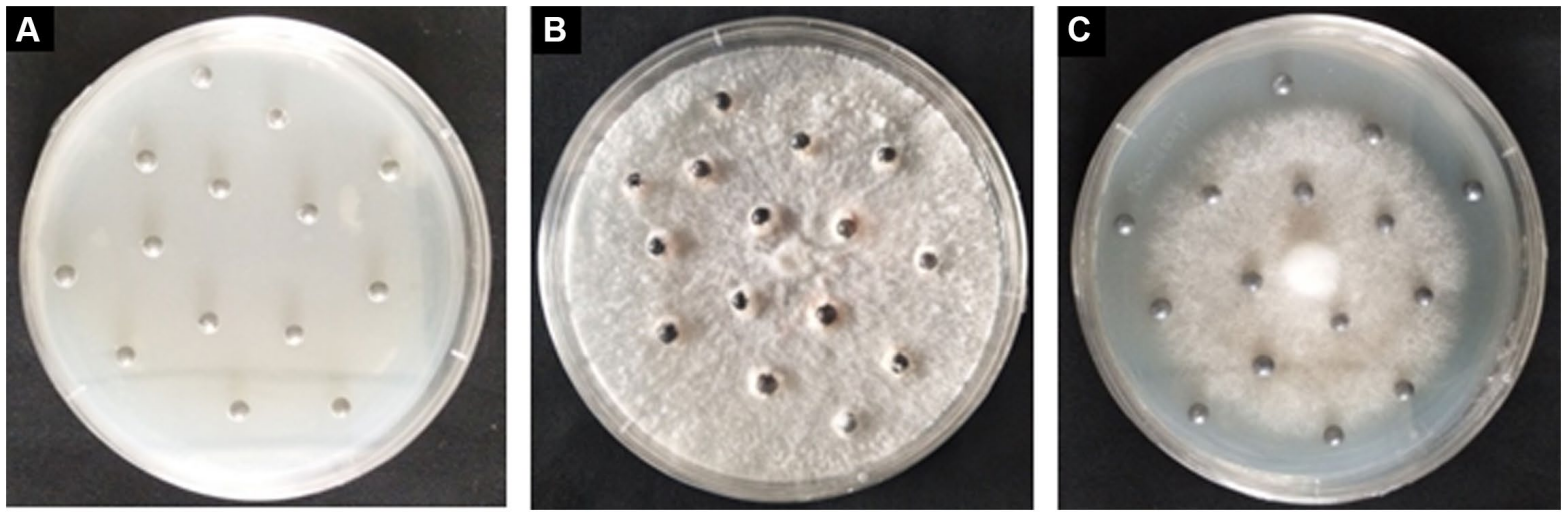
Pb shots on MMN medium without ECM fungi **(A)**; Pb shots on MMN medium with *Suillus grevillei* **(B)**; Pb shots on MMN medium with *Suillus luteus* **(C)**.

Two months after incubation, *S. grevillea* and *S. luteus* grown on the MMN medium with Pb shots (Figure 3). The Pb shots in control medium became milky, while color change of the Pb shots in medium with *S. grevillea* or *S. luteus* was not that obvious.

**Figure 4:**
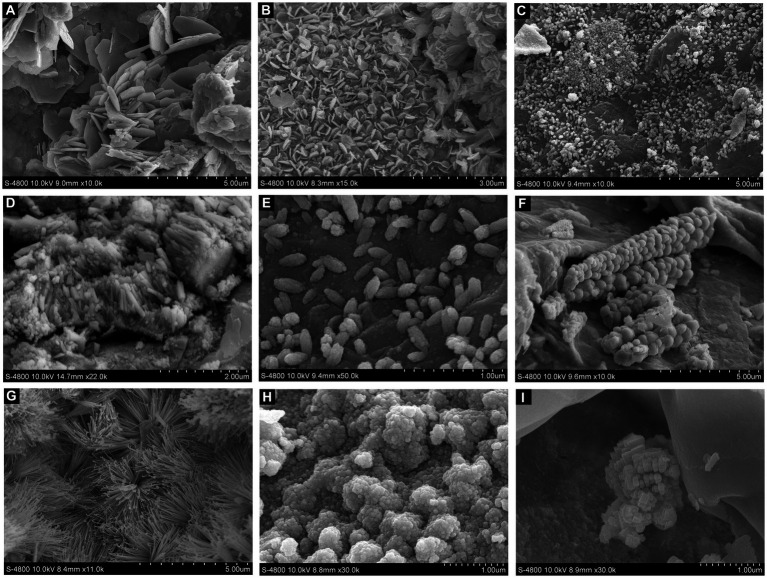
SEM images of the secondary minerals on Pb shot surface at different treatment (**A,B**, non-inoculation; **C–E**, *Suillus grevillei* inoculation; **F–I**, *Suillus luteus* inoculation).

Flakes-shaped secondary minerals were observed on the Pb shots in control medium by SEM (Figures 4A,B). Co-culture of Pb shots and *S. grevillea* resulted in rod-shaped and spindle-shaped secondary minerals (Figures 4C–E). Co-culture of Pb shots and *S. luteus* resulted in botryoidal shaped (Figure 4F), acicular shaped (Figure 4G), globular shaped (Figure 4H), and granular shaped (Figure 4I) secondary minerals.

**Figure 5:**
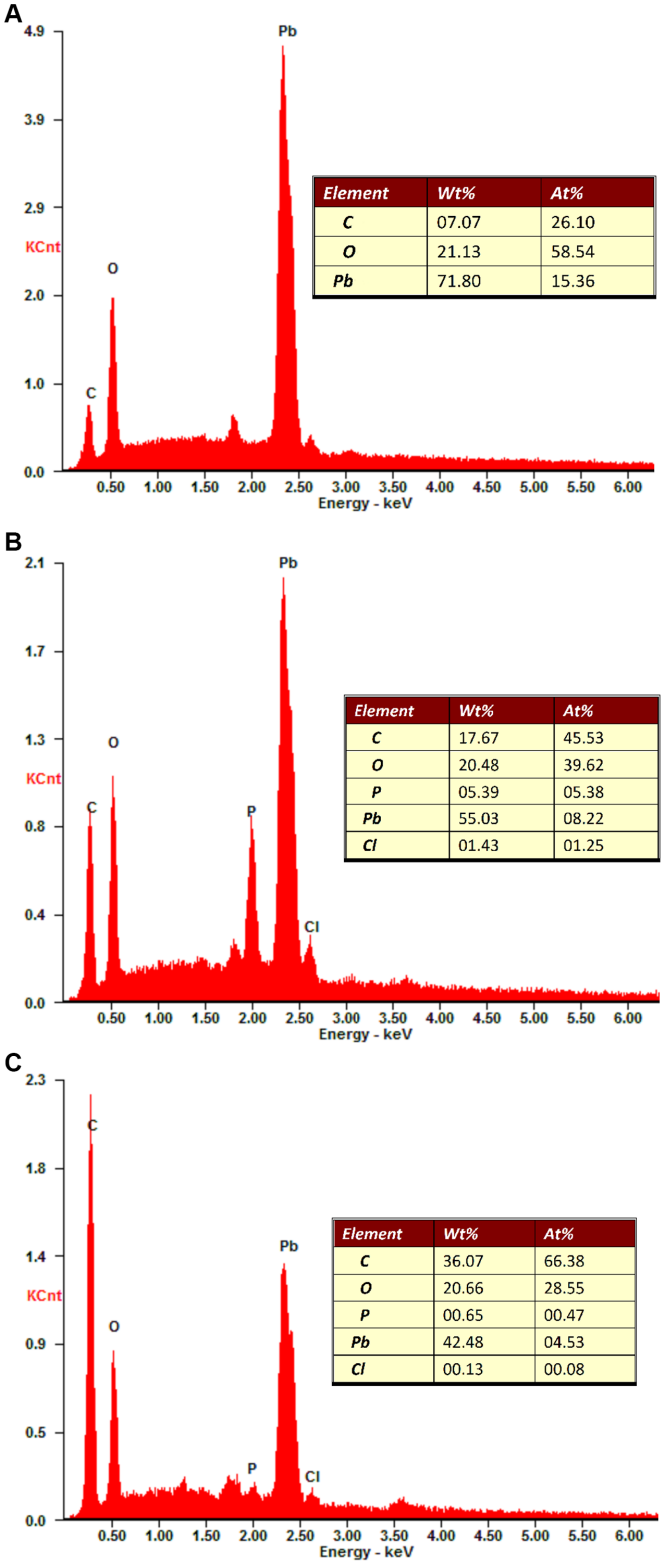
Elemental composition of secondary minerals on the Pb shots after different treatment. wt% and at% refer to weight percentage and atomic percentage of a particular element, respectively. **(A)** The elemental composition of non-inoculation treatment showed that secondary minerals were comprised of C, O, and Pb. **(B)** The elemental composition of *Suillus grevillei* inoculation treatment showed that secondary minerals were comprised of C, O, P, Pb and Cl. **(C)** The elemental composition of *Suillus luteus* inoculation treatment showed that secondary minerals were comprised of C, O, P, Pb, and Cl. The “inset” indicates the weight percentage and atomic percentage of different elements on the Pb shots surface.

Inoculation of two ectomycorrhizal fungi changed the elemental composition of secondary minerals on Pb shots (Figure 5). Carbon (C), oxygen (O), and Pb were the elements on Pb shots in control medium, while phosphorus (P) and chlorine (Cl) appeared along with C, O, and Pb when ectomycorrhizal fungi were in the medium.

**Figure 6:**
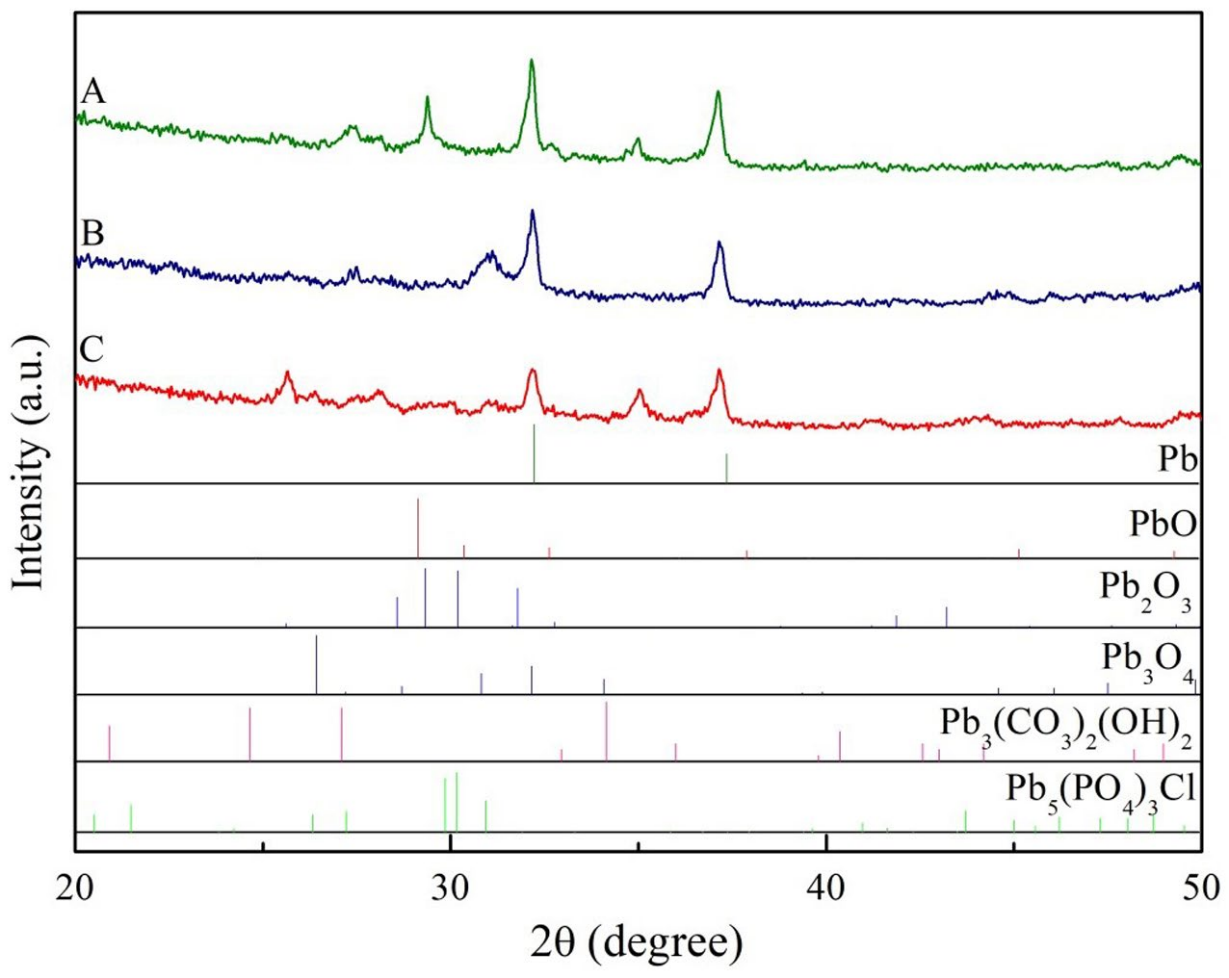
XRD spectrum of the secondary minerals on Pb shots surface at different treatment (A, non-inoculation; B, *Suillus grevillei* inoculation; C, *Suillus luteus* inoculation).

The XRD spectra of secondary minerals at different treatments had abundant spectral line characteristics, indicating that the secondary minerals were crystal. The diffraction peaks of secondary minerals were observed and could be identified as PbO, Pb_2_O_3_, Pb_3_(CO_3_)_2_(OH)_2_ in control treatment and PbO, Pb_2_O_3_, Pb_3_(CO_3_)_2_(OH)_2_, Pb_5_(PO_4_)_3_Cl in two ECM fungi treatment (Figure 6).

## 4 Discussion

Mycorrhizal plants acquire water and mineral nutrients via fungi hyphae, while supply organic nutrients to fungi (Brundrett, 2002; Kaiser et al., 2010; Pena and Polle, 2014). In this study, we observed an increase in the biomass of *P. tabulaeformis* inoculated with *S. grevillea* under both Pb-treated and Pb-free treatment. This improvement caused by *S. grevillea* can be explained by greater mineral absorption (Canton et al., 2016; Sun et al., 2021) and Pb tolerance (Baum et al., 2006). However, the effect of *S. luteus* on the growth of *P. tabulaeformis* was not obvious. Compared with ECM fungi sensitive to heavy metals, the tolerant strains are reported to be more effective in enhancing the metal tolerance and growth of host plants (Adriaensen et al., 2006; Szuba et al., 2017). Thus, we speculated that *S. luteus* exhibit lower Pb tolerance compared with *S. grevillea*, which can be corroborated by the fact that the *S. luteus* colonization rate significantly decreased under Pb Stress (Table 1). Meanwhile, slower growth of *S. luteus* than *S. grevillea* was observed on MMN agar medium with lead shots (Figure 3).

The Pb stress response in both ECM fungi treatments showed improvement in *P. tabulaeformis*, with a decrease in lipid peroxidation (shown by MDA level) and reactive oxygen species (shown by H_2_O_2_ concentration) and a significant improvement in antioxidant defense (shown by SOD, POD, and CAT activity). Our findings support the early studies that discovered the promotion of ECM symbiosis through the rise in antioxidant activities in their host plants against Cb and Al stress (Liu et al., 2020; Sun et al., 2021). In this work, *S. grevillea* inoculation resulted in a considerable rise for carotenes or chlorophylls but not *S. luteus*, which could be related to the different properties of fungi, as mentioned above. This result is supported by the findings of Szuba et al. (2017), with more chlorophylls and carotenes increments of *Populus × canescens* colonized by the Pb-tolerant *Paxillus involutus*. Our results supported the idea that inoculating host plants with ECM fungus considerably increases their Pb tolerance and shields them from heavy metal stress (Szuba et al., 2017).

Pb is a weakly translocated metal. Up to 90% of the Pb absorbed by plants is accumulated in their roots (Kumar et al., 1995; Fahr et al., 2013). The accumulation of Pb in plant roots has been found to be higher than in shoots, particularly in mycorrhizal seedlings where Pb accumulation in roots was significantly elevated compared to non-mycorrhizal seedlings (Table 2). This finding is consistent with previous studies (Bojarczuk et al., 2015; Gu et al., 2017; Hachani et al., 2020; Liu et al., 2020) which have suggested that excess Pb is sequestered through biomineralization in fungus hyphae, often along with colonized roots for analysis. ECM fungi play a crucial role in lead homeostasis within ecosystems by accumulating a substantial amount of lead through their mycelium (Liu et al., 2020; Branco et al., 2022). These mechanisms involve the storage of excessive metals in compartments within cells (González-Guerrero et al., 2008; Ruytinx et al., 2013), and the capture of these metals by proteins or metabolites in ECM fungi (González-Guerrero et al., 2007; Leonhardt et al., 2014). Additionally, excess metals can be exclusion by ECM fungi (Ruytinx et al., 2013; Majorel et al., 2014). Various metal transporters and chelating proteins in mycorrhizal fungi have been functionally studied, providing insight into their role in reducing the harmful effects of excessive environmental metals (Leonhardt et al., 2014; Coninx et al., 2017; Ruytinx et al., 2017; Gómez-Gallego et al., 2019).

To investigate potential interactions between metallic Pb and the ECM fungi, two ECM fungi were incubated with a Pb shot. It was clear from the structural variations between the Pb secondary minerals generated in the presence of the fungus and those formed in the control plates that the fungi had a role in their formation. Subsequently, the particular mineral was identified as pyromorphite (Pb_5_(PO4)_3_Cl). Under general geochemical conditions, pyromorphite is the most stable environmental Pb compound (Ksp = 10^–79.6 ± 0.15^) (Topolska et al., 2016) and pyromorphite deposition has consequently been proposed as a remediation treatment for Pb-contained soil (Miretzky and Fernandez-Cirelli, 2008).

In the natural environment, pyromorphite generation depends on various chemical and biological processes (Rhee et al., 2012). A previous study has demonstrated that *Paecilomyces javanicus* participates in the biomineralization of Pb metal and transforms Pb into pyromorphite and the authors speculated that this phenomenon is linked with microbial transformations of inorganic and organic phosphorus by extracellular acid phosphatases secreted by fungal (Rhee et al., 2012; Liang et al., 2015). This process produced the main ligands (PO_4_^3−^) of Pb, which may contribute to biologically induced pyromorphite formation. The variation of pH could change the dissolution of the Pb and P sources and oxidation–reduction potential (Eh), which affect the efficacy of pyromorphite formation (Chen et al., 2003; Ehrlich and Newman, 2009). Fungi can produce organic acids, which can mobilize metal ions while changing pH (Adeyemi and Gadd, 2005). Similarly, we observed less Pb precipitation in the control medium, which may be attributed to the mobilization of PbO, Pb_2_O_3_, and Pb_3_(CO_3_)_2_(OH)_2_ by organic acids secreted by fungal hyphae.

*In vivo* and *in vitro* growth conditions may alter the nature of the ligands provided by fungal hyphae. However, here we are lacking information on ECM fungi’ effects on Pb biomineralization in the symbiotic state, but Fomina et al. (2005) reported that ECM fungus provides the same ligands of functional groups (e.g., phosphate, carboxylate, sulfhydryl) in both pure culture and ectomycorrhizal association. Thus, it is likely that ECM fungi hyphae in a symbiotic relationship would also provide functional ligands for Pb precipitation.

## 5 Conclusion

Our results revealed a notable, positive impact of inoculating *S. grevillei* and *S. luteus* on *P. tabulaeformis* growth and Pb tolerance, which enhances the establishment of remediation plants under Pb-contaminated soil. Our *in vitro* experiments confirmed that ECM fungi participated in the bio-mineralization of Pb into pyromorphite. As such, our corroboration of pyromorphite formation represents the first discovery of such Pb biomineralization induced by ECM fungi. This observation increases our understanding of the biogeochemical cycle of Pb. ECM fungi induced Pb biomineralization is also relevant to the new approaches in the bioremediation of polluted environments. Predictably, the space between the soil particles, which is unreachable for the roots, is now filled with hyphae of ECM fungi, meaning more extensive and efficient Pb phytostabilization.

## Data availability statement

The original contributions presented in the study are included in the article/supplementary material, further inquiries can be directed to the corresponding authors.

## Author contributions

KC: Conceptualization, Data curation, Formal Analysis, Investigation, Writing – original draft. YL: Investigation, Writing – original draft. MT: Project administration, Supervision, Writing – review & editing. HZ: Conceptualization, Data curation, Funding acquisition, Project administration, Supervision, Writing – review & editing.
